# A Partially Purified *Acinetobacter baumannii* Phage Preparation Exhibits no Cytotoxicity in 3T3 Mouse Fibroblast Cells

**DOI:** 10.3389/fmicb.2016.01198

**Published:** 2016-08-03

**Authors:** Alexandra E. Henein, Geoffrey W. Hanlon, Callum J. Cooper, Stephen P. Denyer, Jean-Yves Maillard

**Affiliations:** ^1^School of Pharmacy and Biomolecular Sciences, Brighton UniversityBrighton, UK; ^2^Department of Molecular Biosciences, The Wenner-Gren Institute, Stockholm UniversityStockholm, Sweden; ^3^Cardiff School of Pharmacy and Pharmaceutical Sciences, Cardiff UniversityCardiff, UK

**Keywords:** bacteriophage, cytotoxicity, *Acinetobacter baumannii*, lactate dehydrogenase, MTS assay, trypan blue, hoechst stain, propidium iodide

## Abstract

A surge in the level and scale of antibiotic resistance has prompted renewed interest in the application of bacteriophages to treat bacterial infections. However, concerns still exist over their efficacy and safety. *Acinetobacter baumannii* phage BS46, a member of the family *Myoviridae*, has previously been shown to be effective in murine models. The cytotoxic effect of this phage was evaluated in mouse fibroblast 3T3 cells using four different assays: trypan blue; staining with Hoechst and propidium iodide; lactate dehydrogenase release; and the MTS assay. The addition of phage concentrations up to 2 × 10^9^ pfu/mL showed little to no impact on the viability of 3T3 cells after 24 h exposure using the different assays. This study demonstrates that phage BS46 is non-cytotoxic to 3T3 cells using four different assays and that appropriate quality assurance protocols for phage therapeutics are required.

## Introduction

There is a resurgence of interest in phage to treat multidrug resistant infections in humans, with some bacteriophage (phage) products entering clinical trials (Rhoads et al., 2009; Wright et al., 2009) or in animal testing (Biswas et al., 2002; Morello et al., 2011). Although the use of phages within a clinical setting dates back to the early part of the 20th Century (Kutter et al., 2010), their use has been primarily confined to the former Soviet Union and Eastern Europe, in part due to concerns about safety and efficacy (Hanlon, 2007). Small numbers of phage preparations have been approved by the U.S. Food & Drug Administration for use as food additives within the food industry for the control of *Listeria monocytogenes* (Bren, 2007) and within the European Union (Anonymous, 2012). The small amount of human clinical trial data that exists in currently available literature has not demonstrated toxicity issues (Merabishvili et al., 2009). However, the potential to induce an immune response regardless of the level of preparation purity is of major concern to those seeking to utilize phage therapy clinically. There are a number of assays available to measure cell cytotoxicity. These often differ in the parameters they measure and have been shown to exhibit different levels of sensitivity (Fotakis and Timbrell, 2006). No standard assay for determining the cytotoxic effect of phage has been reported.

*Acinetobacter baumannii* is an opportunistic human pathogen causing a wide range of infections including wound, urinary tract infections (Antunes et al., 2014) and ventilator-acquired pneumonia (Qureshi et al., 2015). *A. baumannii* is an important multidrug resistant microorganism (Cai et al., 2012; Zarrilli et al., 2013; Antunes et al., 2014), with resistance to colistin being recently reported (Qureshi et al., 2015). The use of phages against this important pathogen is an exciting prospect, but for application in humans, particularly for wound infections, their cytotoxicity needs to be evaluated. Here the *A. baumannii* phage BS46, a member of the *Myoviridae* family was used. It has previously been shown to provide a protective effect in mice when challenged with up to five times the LD_50_ (10^8^ cfu) of *A. baumannii* (Soothill, 1992).

The current investigation sought to determine the cytotoxic effects of a single *A. baumannii* phage and to evaluate different methods of determining cytotoxic effect following the addition of bacteriophage.

## Materials and Methods

All chemicals and reagents were obtained from Fisher Scientific (Loughborough, UK) or Sigma–Aldrich (Gillingham, UK) unless otherwise stated in the text.

Sterile Lambda (λ) buffer was prepared by the addition of 2.5 mL 2% (w/v) gelatin to 6 mL 1 M Tris base at pH 7.2. To this 2.5 g MgSO_4_.7H_2_0 was added and the resulting suspension made up to 1 L with dH_2_O. The resulting solution was then autoclaved at 121°C for 15 min.

### Routine Culture of Bacterial and Bacteriophage Strains

*Acinetobacter baumannii* HER1401 and phage BS46 (Soothill, 1992; Ackermann et al., 1994) were obtained from The Felix d’Hérelle Reference Center for Bacterial Viruses, GREB, Faculté de Médecine Dentaire, Université Laval, Canada.

*Acinetobacter baumannii* HER1401 cultured in Tryptone Soy Broth (TSB; Oxoid, Basingstoke, UK) for 18 h at 37°C under 120 rpm agitation was used to inoculate 100 mL TSB. That was then incubated for 3–5 h at 37°C under 120 rpm agitation until an OD_600_ of 0.5 (∼10^8^ cfu/mL) was reached. Bacterial suspensions were then infected with 1 mL phage suspension (∼10^6^ pfu/mL), statically incubated at 37°C for 15 min and then incubated for a further 18 h at 37°C and 120 rpm.

Following incubation 10 mL chloroform was added and then the suspensions were incubated at 37°C, 120 rpm for 10 min prior to centrifugation at 2500 × *g* for 10 min. The resulting supernatant was then passed through a 0.45 μm syringe filter (Millipore, UK) to produce a crude lysate.

### Production of Concentrated Purified Phage

Prior to use, crude lysates were enumerated and assessed for viability using the agar overlay method (Adams, 1959). To each crude lysate, sodium chloride was added to give a 1 M final concentration, stored on ice for 1 h and centrifuged at 11000 × *g* for 10 min at 4°C. Following centrifugation, 10% (w/v) polyethylene glycol 8000 (PEG 8000) was added to the supernatant and stored at 4°C for 18 h.

Suspensions were centrifuged at 11000 × *g* for 10 min at 4°C, the supernatant discarded, the pellet re-suspended in 11 mL λ buffer and 1 mL chloroform. The organic and aqueous phases were separated by centrifugation at 3000 × *g* for 15 min at 4°C. The organic phase was discarded and the aqueous phase made up to 50 mL with λ buffer and passed twice through 0.45 μm filters. Purified phage preparations were diluted in λ buffer to provide a working suspension of 8 × 10^9^ pfu/mL and enumerated using the agar overlay method as previously described (Adams, 1959) and stored in sterile glass containers at 2–8°C.

Prior to use in cytotoxicity experiments, phages were diluted in λ buffer to yield 2 × 10^9^, 2 × 10^8^, and 2 × 10^7^ pfu/mL and suspensions equilibrated to room temperature.

### Cell Culture

*Acinetobacter baumannii* is a known bacterial pathogen associated with wounds. The embryonic Swiss albino mouse fibroblast cell line ‘3T3(+3)’ ECACC No. 89022402 was used here as a model since fibroblasts are associated with wound healing. 3T3 cells were grown in Dulbecco’s Modified Eagle’s Medium (DMEM) supplemented with 1 g/L glucose, L-glutamine and sodium bicarbonate, 10% (v/v) fetal calf serum (FCS) and 1% (v/v) penicillin and streptomycin. All cells were incubated at 37°C with 5% CO_2_ in a humidified atmosphere.

### Analysis of Bacteriophage Cytotoxicity in 3T3 Cells

#### Preparation of Cells for Use in Cytotoxicity Experiments

Working culture plates were produced by the addition of 5000, 10000, 15000, or 2 × 10^5^ 3T3 cells (depending on the assay type) to wells of sterile Nunclon surface 24-well cell culture plates (Nunc Denmark) into a total volume of 1 mL pre-warmed supplemented DMEM and incubated for 24 h at 37°C in 95% air: 5% CO_2_ in a humidified atmosphere.

In order to remove mouse fibroblast 3T3 cells from 24 well plates, DMEM was removed, 200 μL of trypsin added to each well and plates incubated for 3–5 min at 37°C in a 95% air: 5% CO_2_ atmosphere. Cell suspensions were centrifuged at 400 × *g* for 5 min, the supernatant discarded and the pellet re-suspended in 1 mL fresh DMEM.

### Trypan Blue Exclusion Assay

3T3 cell suspensions and 50 μL purified phage in experimental wells (*n* = 6) were prepared and incubated for 24, 48, and 72 h as before. λ buffer was used as a negative control.

Following trypsinisation, cell viability was assessed using a Trypan blue assay (Strober, 2001). In brief, 20 μL of cell suspension was added to 4 μL 0.4% (w/v) trypan blue solution in phosphate buffered saline (Sigma–Aldrich, Gillingham, UK) and the total number of cells immediately quantified from five squares of a haemocytometer at 100 × magnification using an inverted light microscope (Wilovert standard; Hund Wetzlar, Germany). If the cell density was too high then cells were diluted 1:10 in DMEM prior to imaging. Unstained cells were considered to be alive while blue stained cells were considered to be dead. In all cases, samples from each well were read in quintuplicate on two different occasions.

### Hoechst and Propidium Iodide Staining for Cell Apoptosis

BS46 phages were inoculated to growing 3T3 cells as described above. λ buffer was used as a negative control.

Viable and apoptotic cells were quantified at multiple time points (*t* = 24, 48, 72 h) after phage addition using a modified propidium iodide (PI) staining method (Belloc et al., 1994). In brief, 900 μL of DMEM was mixed with 50 μL of a 1 mg/mL (in 10 mL demineralized water) PI solution and 50 μL of bisbenzimide. Wells were stained with PI and six random fields of view counted using an Axiovert 25 inverted fluorescence microscope (Karl Zeiss Ltd., Welwyn Garden City, UK) with a 420 nm filter. Viable cells were identified by uniform blue fluorescence and apoptotic cells by their fragmented nuclei with either blue or pink fluorescence.

### Lactate Dehydrogenase (LDH) Release

Lactate Dehydrogenase (LDH) values were determined using the CytoTox 96 Non-Radioactive cytotoxicity assay (Promega, Southampton, UK) according to the manufacturer’s instructions. Absorbance at 492 nm was determined in an automatic Titertek Multiskan Plus MKII plate reader (LabX, Midland, ON, Canada), at 492 nm. Absorbance values were adjusted to compensate for the contribution of DMEM and the percentage cytotoxicity calculated as below;

% Cytotoxicity = [Experimental LDH release/Maximum LDH release] × 100.

### Cell Viability Using an MTS Assay

Fifty micro liter of purified phage suspension were added to the experimental wells and incubated for 24, 48, and 72 h as described above. Negative controls containing 50 μL λ buffer and untreated cells were also performed. The number of viable cells was determined using the CellTiter 96 aqueous One Solution Cell Proliferation Assay (MTS; Promega, Madison, WI, USA) according to the manufacturer’s instructions. Absorbance at 492 nm was determined on an automatic Titertek Multiskan Plus MKII plate reader (LabX, Midland, Canada) and expressed as a percentage of non-treated controls to calculate the percentage proliferation status (Carmichael et al., 1987).

### Statistical Analysis

All experiments were performed in triplicate on different days unless otherwise stated in the text.

#### Trypan Blue Exclusion Test

Data were statistically analyzed using a Kruskal–Wallis One way analysis of variance due to the data being non-normally distributed.

#### Apoptotic Assay

Data were statistically treated using a Univariate General linear model and Levene’s test.

#### MTS Assay

One-way between groups ANOVA with Tukey’s *post hoc* test was used to compare cell viability as percentage of controls.

## Results

### Trypan Blue Assay

**FIGURE 1:**
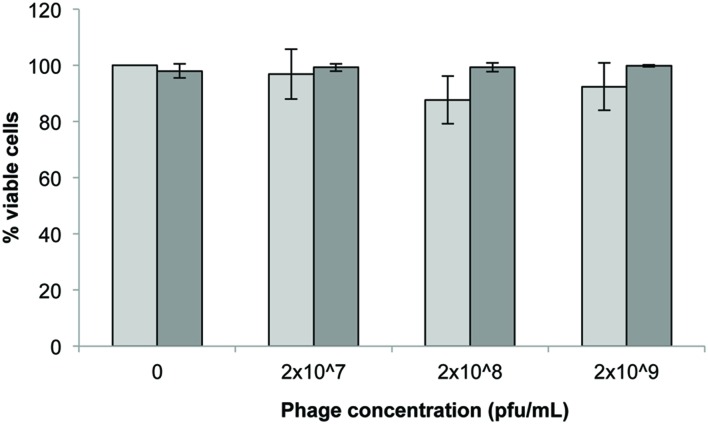
**3T3 mouse fibroblast cell viability following 24 h exposure to *Acinetobacter* phage BS46 in DMEM by trypan blue exclusion assay. 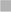: 5 × 10^3^ cells/well; 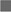: 2 × 10^5^ cells/well.** Data are the mean of 3 replicates ± SD.

The initial concentration of phage showed a non-significant impact (*p* > 0.05) on the viability of 3T3 cells after 24 h exposure (**Figure 1**) with 2 × 10^8^ pfu/mL exhibiting the highest reduction in viability (∼10%). Seeding density (5 × 10^3^ or 2 × 10^5^ cells/well) also had no significant effect on the viability of cells following 24 h exposure to phage (*p* > 0.1 Tukey’s and Kruskal–Wallis).

### Hoechst and Propidium Iodide Staining

**FIGURE 2:**
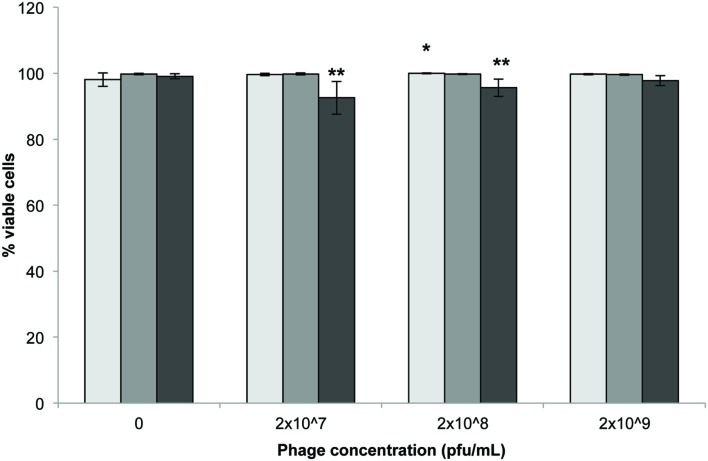
**3T3 mouse fibroblast cell viability following exposure to *Acinetobacter* phage BS46 by Hoechst propidium iodide staining. 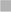: 24, 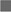: 48 and 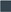: 72 h.** Data shown are the mean of 3 replicates ± SD. ^∗^*p* < 0.05 compared to control, ^∗∗^*p* < 0.001 compared to controls and highest phage concentration (2 × 10^9^ pfu/mL) at 72 h.

Statistically significant reductions in the number of viable cells were observed following 72 h incubation with either 2 × 10^7^ and 2 × 10^8^ pfu/mL compared to untreated controls (**Figure 2**; *p* < 0.001). Incubation time had no significant impact on cell viability (*p* = 0.418).

### LDH Release

**FIGURE 3:**
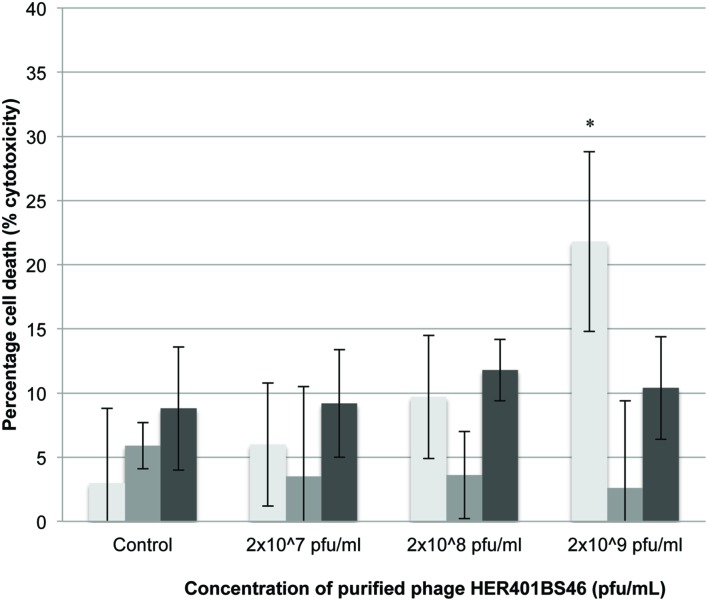
**LDH release of 3T3 mouse fibroblast cells following exposure to *Acinetobacter* phage BS46.** ^∗^*p* < 0.001 compared to the control. 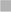: 24, 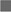: 48, and 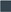: 72 h. Data shown are the mean of 3 replicates ± SD.

Phage addition at varying concentrations resulted in small but not statistically significant increases in cell death compared to untreated samples (between 5 and 10% reduction; **Figure 3**) with the exception of 2 × 10^9^ pfu/mL at 24 h (*p* < 0.001).

### MTS Assay

**FIGURE 4:**
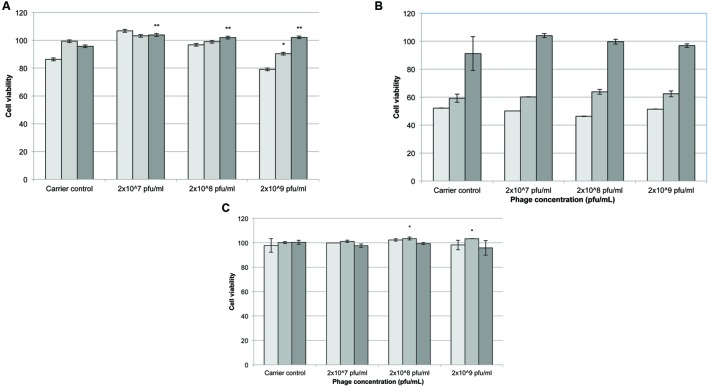
**The effect of *Acinetobacter* phage BS46 on MTS formation of 3T3 cells.** Percentage cell viability was calculated from the untreated cells which represent 100% viability. Carrier control corresponds to cells treated with λ buffer. **(A)**: 24 h, **(B)**: 48 h, and **(C)**: 72 h incubation. 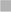 5000 cells/well, 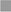 10000 cells/well and 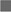 15000 cells/well. ^∗^*p* < 0.05, compared to 2 × 10^7^ pfu/mL; ^∗∗^*p* < 0.05 compared to λ buffer. Data are the mean of 3 replicates ± SD.

Following 24 h exposure to different concentrations of phages (2 × 10^9^, 2 × 10^8^, or 2 × 10^7^ pfu/mL) the number of viable cells appeared to increase at higher initial seeding densities (10000 and 15000 cells/well) compared to controls treated with λ buffer only (**Figure 4A**). At 48 h incubation there was no significant difference between phage samples and λ buffer (**Figure 4B**; *p* > 0.5). After 72 h cells exposed to phages cell viability increased significantly compared to the controls. Cells seeded at 10000 cells/well remained more viable (**Figure 4C**; *p* < 0.05) when exposed to 2 × 10^9^ and 2 × 10^8^ pfu/mL phage, compared to λ buffer and untreated (DMEM only) cells.

## Discussion

Bacterial cell products can have both direct and indirect cytotoxic effects on cells in the stationary phase (Perfetto et al., 2003), particularly through the action of bacterial endotoxins which affect the expression of adhesion molecules, inflammatory responses (Tang et al., 2011), cytokine release (Perfetto et al., 2003; Tardif et al., 2004), or the release of bacterial toxins (Los et al., 2013). However, little information exists in the current literature on the effects of the direct addition of therapeutic bacteriophages on immortalized cell lines such as 3T3 fibroblasts.

In the current investigation, a partially purified *A. baumannii* phage preparation was not cytotoxic to 3T3 mouse fibroblast cell line, although some assay dependent differences in viability were observed. No statistically significant differences were seen between 3T3 cells treated with phage and untreated controls, corresponding with similar investigations performed by Merabishvili et al. (2009) against human neonatal foreskin keratinocytes when assessed by trypan blue exclusion. While patient derived cells will offer the closest analog to whole organism testing *in vitro*, the use of such cell types comes with associated cost and ethical considerations as well as increased variation between multiple donors. Although no substitute for *in vivo* testing, the use of immortalized cell lines would decrease the overall research cost of preclinical studies and enable standardization between laboratories, allowing for better comparative testing to be performed. In addition fibroblasts were used in an attempt to understand the effect of phage on skin or wounds, recognizing that *A. baumannii* is an important wound pathogen.

Although no cytotoxic effect was observed with all the assays tested, the assessment of cellular viability with the MTS assay suggested that phages provided a positive effect on cell viability. This observation supports that of Chung et al. (2010) who reported that genetically engineered M13 bacteriophage improved cell attachment under specific experimental conditions. Although no direct cytotoxic effect was observed, the addition of phages has been shown to induce pro-inflammatory cytokines such as IL-10 and IFN-γ (Dąbrowska et al., 2014; Park et al., 2014). Majewska et al. (2015) reported a weak antibody production in mice following T4 phages ingestion. However, this is dependent on phage type and the protein composition of individual phages (Dąbrowska et al., 2014) and would be of particular importance in non-topical applications that would result in the increased exposure of phages to the immune system (Hodyra-Stefaniak et al., 2015). Here we have used a partially purified phage and it is possible that bacterial debris were present in the preparation. However, the phage preparation used here did not show any cytotoxicity overall. The endotoxin level of any phage product would need to be measured for regulatory purposes before commercialization. A number of methods to reduce endotoxin levels have been described: a single round of PEG precipitation and centrifugation has been shown to remove up to 88% of endotoxins from a bacteriophage preparation (Branston et al., 2015). However, additional purification stages such as ultracentrifugation in a CsCl gradient or chromatographic methods will further reduce impurities (Boratyński et al., 2004).

The current investigation sought to assess the cytotoxicity of a purified preparation of *A. baumannii* phage BS46 using four separate methods. Some discrepancies in the results were observed, notably at high phage concentrations, with the PI and MTS assay showing some small but statistically significant reduction in cell number and the LDH assay showing no significant differences in cell viability. The LDH assay presented the highest variability in results as indicated by the large error bars. It measures the activity of the oxidoreductase of LDH, a stable enzyme, released from damaged cells in the culture medium. Variability in activity, which has been reported in other applications (Bopp and Lettieri, 2008), may be cause by differences in cell seeding. Such variability in results was not noted with the other assays performed. Both the Trypan blue exclusion assay and the Hoechst and PI assays require microscope based analysis which may limit their usefulness although high throughput protocol could potentially be increased using flow cytometry. The MTS assay gives indirect measurement of viability [requires metabolism of MTS (3-(4,5-dimethylthiazol-2-yl)-5-(3-carboxymethoxyphenyl)-2-(4-sulfophenyl)-2H-tetrazolium] to formazan and not a measurement of cell damage. This indirect measurement based on cell metabolism may explain why the MTS assay was the only one that showed an increased in cell viability. For developing a product for phage therapy, for which a high number of samples need to be processed, the high throughput trypan blue assay may be the most practical protocol to use. It is thus important to consider the method used to assess the cytotoxic effects of phage preparations and highlights the pressing need for standardized phage testing protocols.

The purified phage suspension exhibited little toxicity and this result is encouraging for the treatment of topical infections with phages. For non-topical applications, the assessment of cytotoxicity could be complemented with the determination of immune-stimulatory capacity (Merabishvili et al., 2009; Szermer-Olearnik and Boratyński, 2015).

## Author Contributions

Conceived and designed experiments: AH, GH, SD, J-YM. Performed experiments AH. Analyzed data: AH, GH, SD, J-YM. Wrote the manuscript: CC, J-YM, GH, SD.

## Conflict of Interest Statement

The authors declare that the research was conducted in the absence of any commercial or financial relationships that could be construed as a potential conflict of interest.
